# A focus group study to assess perspectives of patients with irritable bowel syndrome on human milk oligosaccharides and lifestyle insights

**DOI:** 10.1007/s00394-025-03719-5

**Published:** 2025-05-30

**Authors:** Patricia Sanz Morales, A. Wijeyesekera, M. D. Robertson, J. Kennedy, G. Major, C. L. Boulangé, G. R. Gibson

**Affiliations:** 1 Department of Food and Nutritional Sciences, University of Reading, Whiteknights, Reading, Berkshire, RG6 6AH UK; 2 Department of Nutrition, Food and Exercise Sciences, Faculty of Health & Medical Sciences, University of Surrey, Guildford, GU2 7XH UK; 3 Nestlé Institute of Health Sciences, Nestlé Research, Société Produits Nestlé, Route du Jorat 57 Vers-chez-les-Blanc 1000, Lausanne 26, Switzerland

**Keywords:** Focus group, Human milk oligosaccharides, Irritable bowel syndrome, Quality of life

## Abstract

**Objective:**

To explore the impact of Irritable Bowel Syndrome (IBS) on quality of life (QoL) measures, with specific focus on symptoms experienced and their impact upon diet, lifestyle, and mood. Insights into triggers of IBS flare-ups and the potential barriers to the use of prebiotics, in particular human milk oligosaccharides (HMOs), will be used to inform the design of a prospective human dietary intervention trial.

**Method:**

Five virtual focus groups were held between March 2022 and January 2023. Thirteen females and eleven males were recruited around Berkshire, UK and through social media to attend a single, same-sex focus group. Thematic analysis of transcripts was undertaken. Themes were organised using a semantic coding tree.

**Results:**

Low QoL in IBS was apparent. Triggers which resulted in worsening symptoms or flares discussed by the groups were all consistent with well-recognised triggers for IBS in the literature and clinical practice. Few participants (6 out of 24) had tried biotic-based therapies (e.g. probiotics, prebiotics) and knew little to nothing about HMOs.

**Conclusions:**

Individuals with IBS could be made more aware of novel therapy options such as prebiotic HMOs which may improve IBS symptoms.

## Introduction

Irritable Bowel Syndrome (IBS) is a highly prevalent disorder of gut-brain interaction (DGBI) with significant negative impact on quality of life and high healthcare costs [1]. Although the prognosis of IBS is not progressive, it is a disorder that poses a considerable burden on individuals and society. Patients present with chronic abdominal pain and an altered bowel habit, frequently accompanied by bloating and distension. Often, IBS will affect patients for life, with flares of activity followed by unpredictable periods of remission [2]. Incidence commonly peaks in the third and fourth decades of life, with females more likely to be affected [3]. Symptomatic IBS has been associated with a significant economic impact due to loss of work productivity and absenteeism [4, 5].

IBS has been associated with alterations at the mucosal gut microbiota-host interface that may induce symptoms. Therefore, microbiota-targeted interventions may benefit people with IBS by positively modulating the gut microbiota [6]. Both prebiotics and probiotics have been trialled as therapy options in IBS [7].

Human milk oligosaccharides (HMOs) are complex carbohydrates, with a core structure of lactose, found in human breastmilk [8]. The majority of ingested HMOs reach the large intestine intact, where they provide selective substrates for gut bacteria [9]. They are candidate prebiotics that specifically stimulate *Bifidobacterium* spp. and are considered to be a defining factor in shaping infant health during early postnatal life. HMOs have recently gathered interest in the context of adult gut conditions, such as IBS [10, 11]. Their potential application for IBS has been studied in two trials, with promising results [10, 12].

Focus groups are a form of group discussion on a central theme, led by a moderator or facilitator. They are well suited to capturing participants’ knowledge, attitudes and experiences on a particular topic [13]. Virtual focus groups are feasible, offer a wider geographical catchment, are more convenient for participants, and are lower in cost than traditional focus groups [14]. Previous qualitative studies have used focus groups to study patient perspectives on living with IBS [15], healthcare delivery [16] and their attitudes towards probiotics [17]. No study to date has explored the opinions of people living with IBS on prebiotics or HMOs.

## Methods

### Participant recruitment

Volunteers with IBS were recruited via social media (Facebook, Instagram, and Twitter/X), recruitment posters on online IBS support groups, and by distributing posters around Reading, Berkshire, United Kingdom. This study was given favourable ethical opinion by the University of Reading Research Ethics Committee School of Chemistry, Food and Pharmacy, Study Number 11/2022.

### Screening

English-speaking volunteers aged 18 years and over with a diagnosis of IBS made in primary or secondary care were sent an information sheet and gave consent to participate. Once this was received by the researcher, participants were sent an online invitation to a focus group.

Additionally, participants completed a short demographic survey through the Research Electronic Data Capture platform (REDCap) [18], indicating their IBS subtype, sex and age (Table 1).

**Table 1:** Questions used in the focus group to semi-structure interviews

Part 1: IBS quality of life
How long have you been living with IBS?
What has your experience with IBS been like so far? Does having IBS affect your lifestyle?
Have you identified any triggers of IBS flare-ups?
Have you tried any therapies that have or haven’t worked for you?
Anything else you would like to discuss before moving on to part 2?
Part 2: Views on human milk sugars and new therapy options for IBS
Have you ever heard of human milk sugars? What comes to mind when you hear this term?
Would you be willing to try a new treatment option for IBS such as this?
If so, what format and duration?

### Group assignment

Focus groups were sex-defined and included participants with different IBS subtypes. No randomisation was conducted. A maximum of six participants were admitted per group. Five focus groups were carried out with a median duration of 65 ± 23 min.

### Conduct of the focus groups

Focus groups were held online via Microsoft Teams and moderated by a single principal investigator (PI). The groups were three to six participants in size and same-sex, allowing for a comfortable discussion session [19]. Sessions were recorded and transcribed automatically. No field notes were made during the focus groups. Broad transcripts were proofed by the PI prior to thematic analysis.

An initial script with questions and prompts were constructed alongside experts in the field, and used to maintain a low session structure inter-variability (Table 1). The questioning route followed a traditional “opening, introductory, transition, key questions, ending questions” format [20]. Although a script was used, sessions were semi-structured, and participants were encouraged to interact with each other [20]. Each session was under 120 min long and had two parts, the first aimed to explore IBS quality of life and the second studied IBS therapies and views on HMO (Table 1).

Participants were compensated with a voucher and were permitted to provide feedback after completing the session. All information-sensitive data was stored in password-protected devices, accessible only by the research team.

### Analysis

**Fig. 1:**
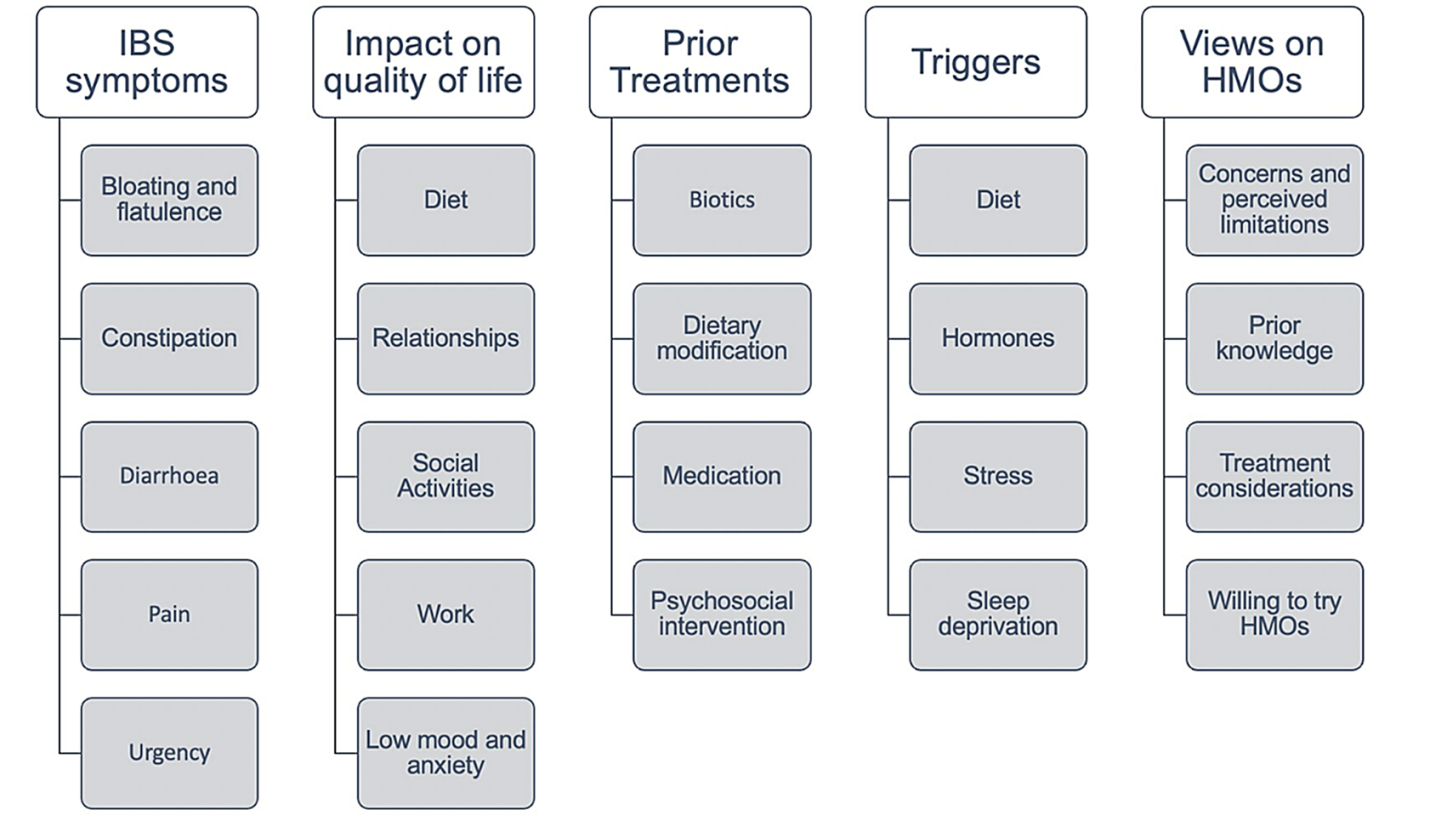
Coding tree showing deductive codes used in NVIVO for semantic thematic analysis

Qualitative analysis was performed using the software package NVIVO 20.6.1.1137. Transcripts were coded using a deductive coding tree (Fig. 1), put together by a multi-disciplinary team including the PI (a registered associate nutritionist) and a gastroenterologist. Coding was performed independently by the coders prior to collaborating on the final coding tree to minimise bias. Codes were organised into five broad themes. Semantic analysis was used throughout, in line with previous studies [21]. Illustrative quotes are provided to supplement narrative descriptions.

## Results

### Participants

24 participants were recruited; 13 female and 11 male (Table 2). Ten participants had diarrhoea-predominant IBS (IBS-D), six had constipation-predominant IBS (IBS-C), seven had mixed-type IBS (IBS-M) and one unclassified IBS (IBS-U). The median age of all participants was 26.5 with an interquartile range of 18.5.

**Table 2:** Focus group composition in chronological order. *Median ± interquartile range (IQR). IBS-M (mixed subtype), IBS-D (diarrhoea predominant), IBS-C (constipation predominant), IBS-U (unclassified IBS)

Focus group	Gender	*n*	IBS subtypes	Median age*
1 (March 2022)	Female	5	2 IBS-M, 1 IBS-D, 1 IBS-C, 1 IBS-U	43 ± 27.5
2 (March 2022)	Female	3	2 IBS-M, 1 IBS-C	21
3 (May 2022)	Female	5	1 IBS-M, 2 IBS-D, 2 IBS-C	26 ± 18.0
4 (January 2023)	Male	6	1 IBS-M, 4 IBS-D, 1 IBS-C	27 ± 8.0
5 (January 2023)	Male	5	1 IBS-M, 3 IBS-D, 1 IBS-C	27 ± 16.5

### Symptoms and triggers

Symptoms of IBS highlighted by the participants were consistent with Rome IV criteria for diagnosis of IBS [22]. As would be expected, most participants with IBS-D discussed diarrhoea as a troublesome symptom, whereas the majority of participants with IBS-C describe constipation, and participants with IBS-M described both diarrhoea and constipation.

In addition, the triggers which resulted in worsening symptoms or flares discussed by the groups are all consistent with well-recognised triggers for IBS in the literature and clinical practice and all participants identified dietary triggers which exacerbated their symptoms [23]. Stress seemed to be more of a significant trigger in the IBS-M group, and a small but significant number of the IBS-C and IBS-M group highlighted hormonal changes such as during their menstrual cycle or the menopause as an important trigger. Three participants described their symptoms worsening with sleep deprivation (Table 3).

**Table 3:** Symptoms and triggers highlighted by participants, with prior IBS treatments tried and illustrative quotes

(% total (*n*/subtype))	IBS-D	IBS-C	IBS-M	Illustrative quote
**Symptoms reported**				
Bloating and flatulence	12.5% (3/10)	8.3% (2/6)	25% (6/7)	“Obviously you’re just feeling full and bloated and you don’t want to eat”.
Constipation	8.3% (2/10)	12.5% (3/6)	20.8% (5/7)	“I don’t move my bowels for like 4–5 days in a row and then that becomes problematic”.
Diarrhoea	29.2% (7/10)	4.2% (1/6)	20.8% (5/7)	“Having attacks of diarrhoea after I’ve been to a restaurant and had a big special meal out and it was an absolute pain”.
Pain	25% (6/10)	12.5% (3/6)	25% (6/7)	“I have awful cramps all the time if I don’t take the medication, but they would come in weak spasms now. So before where I would just have it now and again and it would come, and it would go and it would be all the time… I would constantly have an upset tummy. It would constantly be pain.”
Urgency	16.7% (4/10)	0% (0/6)	8.3% (2/7)	“Uhm, sudden and urgent diarrhoea. I had accidents, outs in the car, in the shops. It’s horrendous.”
**Triggers identified**				
Diet	41.7% (10/10)	20.8% (5/6)	29.2% (7/7)	“I’ve also added six months of dairy free and it didn’t make a blind bit of difference and I can eat onions one day and the next it can trigger an attack like nobody’s business”.
Hormones	0% (0/10)	8.3% (2/6)	4.2% (1/7)	“I noticed sort of the IBS symptoms really sort of flaring up because before that I sort of just thought like it always seemed to be around when I was due on my period as well that it would flare up a lot.”
Stress	12.5% (3/10)	12.5% (3/6)	20.8% (5/7)	“I think for me, emotional stress is a bigger trigger than work stress. If they depend on the person, but I’m quite confident on what I’m doing and even if I’m stressed at work, I know it’s work. I can, you know, just shut down. I’m lucky on that aspect. But emotional stress, like parents or not being able to help a friend or, you know, being stuck on something that for me triggers being scared, also triggers.”
Sleep deprivation	0% (0/10)	4.2% (1/6)	8.3% (2/7)	“Going out and like a lack of sleep really, really messes me up”.
**Prior treatments tried**				
Dietary modification	29.2% (7/10)	20.8% (5/6)	25% (6/7)	“I’ve tried everything really. I even tried the FODMAP…And that’s not worked…I haven’t found anything.”
Biotics	4.2% (1/10)	4.2% (1/6)	16.7% (4/7)	“I do every day take a prebiotic or probiotic. And a digestive enzyme to help with the bacteria, especially if you’ve had like bouts of antibiotic that can just wipe out the balance in your gut.”
Medications	12.5% (3/10)	12.5% (3/6)	25% (6/7)	“I just remember this doctor diagnosed me with IBS and with acid reflux, and she gave me a couple of the like lansoprazole and something else, and mebeverine and that was kind of it. And I I think it’s another drug that’s Rennie/renetolin or something similar to lansoprazole, cycle through those and ended up on lansoprazole because she was just like there’s nothing else you can try this is it.”
Psychosocial and physical interventions	25% (6/10)	12.5% (3/6)	25% (6/7)	“But unfortunately, I haven’t had anything that’s worked. I’ve tried yoga. And had multiple rounds of like CBT therapy…what else, I’ve done like painting. Just like trying to do self-care things”.

### Prior treatments

**Fig. 2:**
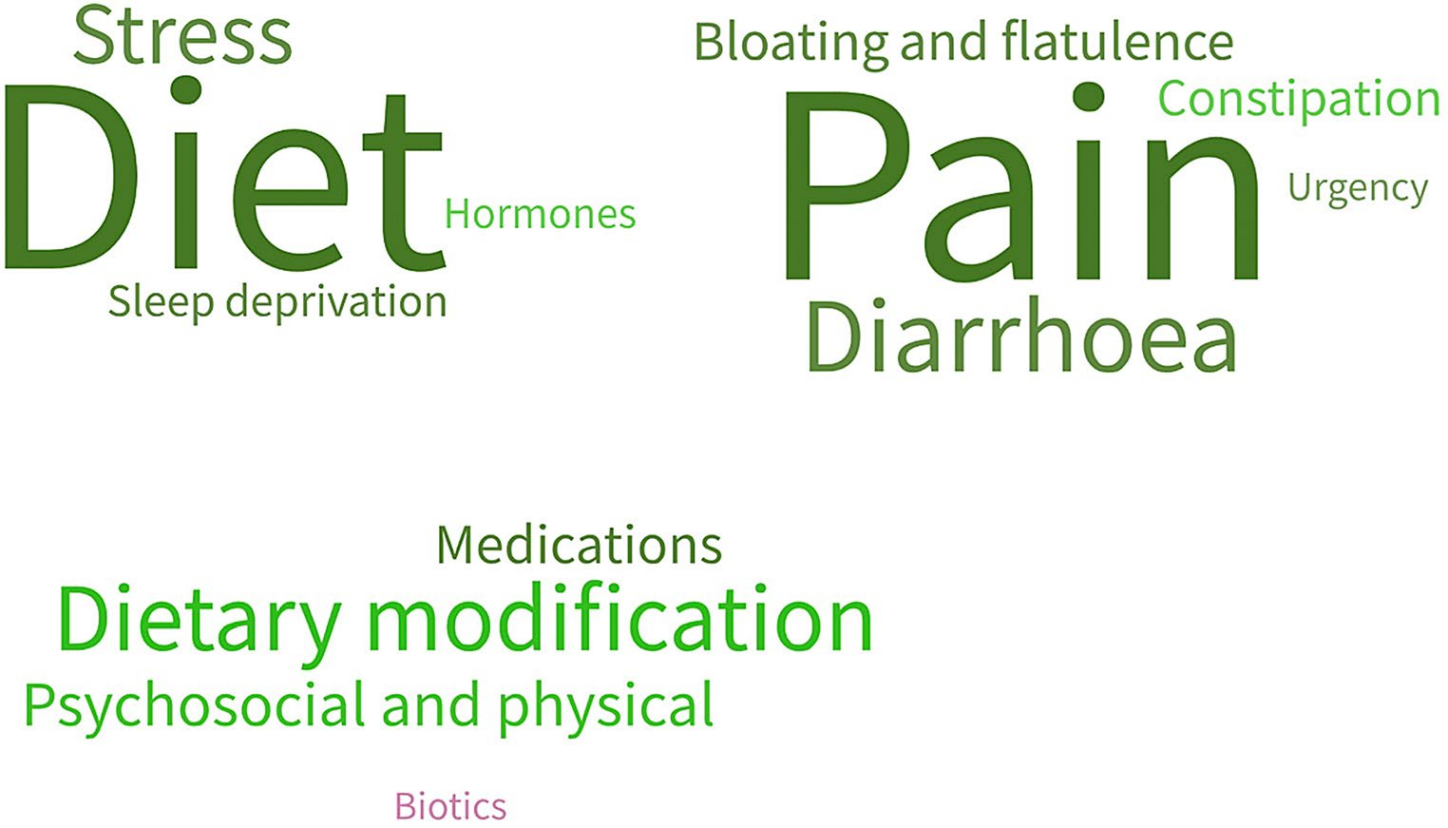
Word cloud demonstrating relative weighting of triggers, symptoms and prior treatments

It was universal that participants had tried at least one modality of intervention for their IBS. The most common was dietary modification. Over half had taken medications, while over half had used psychosocial interventions (such as mindfulness or cognitive behavioural therapy) or physical interventions (such as exercise or yoga). Only six of twenty-four participants described having tried prebiotics, probiotics or fermented foods to help with their symptoms (Fig. 2).

Although a few participants reported some relief from particular interventions, the overarching theme was that most treatments tried worked only partially, or not at all (Table 3).

### Impact on quality of life

Five key domains were apparent when discussing the impact of IBS on individuals’ quality of life: social activities, diet, relationships, work and mental health.

Regarding social activities, participants reported *“segregating themselves…because of this disorder”*, *“being restricted from doing lots of things”* and being *“reluctant to make plans”*.

Unsurprisingly, given the importance of dietary triggers, participants felt that restrictions placed on their diet by the condition impacted on their quality of life, especially when combined with socialising:

I just have to be careful what I eat, which is really horrible, especially when you’re with friends and they wanna go to [a pizza chain] and…I know if I eat this, I’m gonna be paying for it later.

Developing intimate relationships was a challenge for some participants: *“Nobody wants to know about this. There is nothing nice about it.”* Having IBS made some participants nervous about meeting a potential new partner.

Regarding work, participants reported significant impact on their employment and academic work if they were still in education, missing out on shifts at work or taking time off studying. In addition, unpredictability of symptoms posed particular issues with travel.

e.g. “*Uhm*,* sudden and urgent diarrhoea. I had accidents*,* outs in the car*,* in the shops. It’s horrendous. Uhm lockdown was actually nice for me because I didn’t have to go anywhere*.”

Finally, several participants reported concurrent symptoms of fatigue, low mood and anxiety, and two pointed out a negative feedback loop between anxiety symptoms and IBS: *“And it’s that you get stressed*,* so it’s worse and it’s worse because you get stressed”.*

### Prior knowledge and concerns regarding HMOs

Prior knowledge regarding HMOs, and prebiotics in general was minimal across the participants. When described as ‘human milk sugars’, several participants identified HMOs as a constituent of breast milk, but only one participant with a background in biomedical science knew their prebiotic potential.

Participants expressed some concerns about HMOs as a novel therapeutic. There were concerns regarding the provenance of HMOs, including whether the product was *“natural”*, and whether it was derived from human breast milk:

I don’t wanna take it from the baby…if it’s, you know, made in the lab and, you know, it’s not harming anyone else or depriving any babies.

The cost of HMOs to the end user came up as a concern, and whether the price would mean they would still be accessible as treatments to other patients on reduced incomes.

Finally, participants were also concerned regarding whether HMOs had any side effects which may limit their use, and whether they may be addictive or lead to dependency.

### Treatment considerations and willingness to try HMOs

Treatment considerations regarding dosing, formulation and duration of therapy were coded separately to concerns regarding the above patient concerns and scepticism regarding HMOs.

Participants had differing views regarding formulation of the final product. A few expressed that they would be happy with a pill or powder, but the thought of being able to add the product to food was particularly appealing. Properties important to participants included taste (i.e., that the product would not have an offensive taste), and whether there were restrictions about when the product could be taken:

One thing that would be important to me would be it not having restrictions in terms of what you can and can’t eat like right after taking it and before …, and I find that really difficult because I do eat six times a day strictly because I need to get the nutrition in some way and I can only tolerate small meals.

If you were able to like not have it on an empty stomach then that would be really beneficial.

On the whole, participants preferred a duration of therapy of weeks rather than months or long term.

## Discussion

There has been increased scientific attention in understanding the nature of gut conditions from the perspective of patients. Previous efforts to achieve this have been through physician assessments or clinical surveys where questions are developed by study investigators [16]. These methods are insufficient when assessing illness experience such as QoL and severity, or views on potential new therapeutic options such as prebiotics or HMOs. In this study of adults with IBS, insight was gained on lifestyle and views on potential new therapy options with prebiotics and HMOs. Five overarching themes were identified from the semi-structured interviews: IBS symptoms, impact on QoL, prior treatments, triggers and views on HMOs.

Conducting focus groups online supported the inclusion of English-speaking IBS participants from diverse regions in the UK, improving accessibility and allowing participants to attend from their own home [14]. Moreover, same-sex groups allowed for more comfortable, open discussions. For instance, women linked IBS symptoms and flare-ups to the menstrual cycle or the menopause [24].

Possible limitations highlighted in the literature include reduced recruitment of older patients and those with poor internet use, although information on quality of data between in-person and virtual groups is lacking [25]; indeed in this study only one participant over 60 was included. Studies on quality of data between in-person and virtual groups are lacking, but depth of response and participant engagement did not seem to be compromised in this study. Also, availability of participants with varying schedules meant groups varied in size, IBS subtypes and age (Table 2).

Previous studies have highlighted the heavy burden of IBS on QoL [15, 26]. Study participants expressed frustration with existing treatment options for IBS being suboptimal and conflicting. The NICE guidelines for management of IBS in adults highlight dietary and lifestyle advice, and include a recommendation of probiotics which should be taken at least for four weeks while monitoring their effect [27]. Specific probiotic strains have already been marketed to improve digestive health, although individuals with IBS have many unanswered questions about their use [17, 28]. Two exploratory human trials have been published to date investigating the use of HMOs to alleviate IBS symptoms [10, 12]. The novelty of this focus group study, is the qualitative investigation of patient opinion on the potential use of HMOs to treat IBS, which is encouraging to researchers looking to perform more research within the field.

Moreover, fatigue is a common symptom experienced by patients with chronic disease, and disorders of gut-brain interaction such as IBS are no exception [29]. Although not part of the diagnostic criteria for IBS, a number of the participants described fatigue which impacted on their activities of daily living. This identifies fatigue as a possible patient-reported outcome measure for future intervention trials in IBS.

Although participants had minimal prior knowledge of HMOs, there were some legitimate concerns, outweighed by willingness to try this potential new therapeutic option. Once participants were given a definition of HMOs, including their manufacturing process using engineered cells of *E. coli*, most found HMOs appealing because they were perceived as more “natural” and accessible than pharmaceutical drugs and understood them to have little or no risk of side effects. These results complement previous findings on patients’ perspectives on probiotic use [17].

Findings from this study are relevant to clinical practice since gastroenterologists and general practitioners are uniquely positioned to recommend prebiotics and HMOs in IBS treatment. We believe it is important that healthcare professionals in particular stay at the cutting edge of gut microbiota modulating therapy and are aware of what their patients are thinking.

## Conclusions

This study suggests IBS sufferers are interested in using HMOs to improve their gut health and IBS symptoms, although they have unanswered questions about their efficacy. The increased popularity and availability of HMOs to treat adult gut conditions should lead to further exploration of their use by individuals with IBS, and clinicians should be better prepared to discuss prebiotics in general as well as the specific use of HMOs as potential IBS therapies in the near future. These results should also be taken into consideration in the design of prospective clinical trials investigating potential new IBS dietary therapies with HMOs.

## Data Availability

Data will be made available upon reasonable request.

## Abbreviations

DGBI: Disorder of Gut-Brain Interaction
HMOs: Human Milk Oligosaccharides
IBS: Irritable Bowel Syndrome
IBS-C: Constipation-predominant IBS
IBS-D: Diarrhoea-predominant IBS
IBS-M: Mixed-type IBS
IBS-U: Unclassified IBS
IQR: Interquartile Range
NICE: National Institute for Health and Care Excellence
PI: Principal Investigator
QoL: Quality of Life
REDCap: Research Electronic Data Capture platform
UK: United Kingdom
